# Draft Genome Sequence of *Clostridium ultunense* Strain Esp, a Syntrophic Acetate-Oxidizing Bacterium

**DOI:** 10.1128/genomeA.00107-13

**Published:** 2013-03-28

**Authors:** Shahid Manzoor, Bettina Müller, Adnan Niazi, Erik Bongcam-Rudloff, Anna Schnürer

**Affiliations:** a Department of Animal Breeding and Genetics Science, Swedish University of Agricultural Science, SLU Global Bioinformatics Centre, Uppsala, Sweden; b Department of Microbiology, Swedish University of Agricultural Sciences, Uppsala BioCenter, Uppsala, Sweden; c University of the Punjab, Lahore, Pakistan

## Abstract

*Clostridium ultunense* strain Esp belongs to the functional group of syntrophic acetate-oxidizing bacteria (SAOB), which have been identified as key organisms for efficient biogas production from protein-rich materials. Genome analysis and comparative genomics might aid us to define physiological features that are essential for maintaining this particular syntrophic lifestyle.

## GENOME ANNOUNCEMENT

*Clostridium ultunense* strain Esp was isolated from sludge of an upflow mesophilic anaerobic filter treating wastewater from a fish meal-producing factory. It is strictly anaerobic, motile, and spore forming, and the rod-shaped cells tend to form chains (1). It shares 99% 16S rRNA gene sequence identity to the type strain of *C. ultunense*, isolated from a mesophilic triculture by the same laboratory (2). *C. ultunense* was shown to grow heterotrophically on a small number of low-energy substrates, producing acetate as the only end product through the Wood-Ljungdahl pathway, and has therefore been allocated to the group of homoacetogens (1–3). However, cocultivation experiments with methanogenic archaea indicated syntrophic acetate-oxidizing (SAO) capability (1, 3, 4). It has been shown by genetic and enzymatic studies that syntrophic acetate-oxidizing bacteria (SAOB) most likely reverse the Wood-Ljungdahl pathway and oxidize acetate to hydrogen and carbon dioxide when growing in syntrophy with methanogens (3–5). Genome analysis and comparative genomics, including the recently sequenced and annotated genome of the mesophilic SAOB *Tepidanaerobacter acetatoxydans* strain Re1 (6), might aid us to define general features that are essential for maintaining a syntrophic lifestyle and to answer questions concerning regulation, energy conservation, and electron transfer mechanisms. This knowledge will enable us to further understand the mechanisms triggering SAO in different environments, to monitor the activities of known SAOB, and to find new isolation strategies.

The whole-genome shotgun sequencing of *C. ultunense* strain Esp was performed by using the Ion Torrent PGM system, generating a total of 2,631,078 single-end reads with an average length of 206 bp. Low-quality and short reads were filtered, and the remaining 2,583,278 (98.1%) reads were assembled with Newbler 2.8 by the *de novo* assembly method. A total of 2,480,436 reads (96.02%) were aligned to produce 281 scaffolds with an *N*_50_ of 38,263 bp. The average length of contigs was 21.8 kb. The final approximate coverage for these contigs was about 18×. Open reading frames (ORFs) were predicted by MaGe (Microbial Genome Annotation & Analysis Platform). rRNAs were predicted by using RNAmmer (7), and tRNAs were identified with tRNAscan-SE 1.21 (8). A total of 6,446 ORFs were predicted, including 6,296 protein-coding sequences, 103 tRNAs, five copies of 5S rRNA, and one copy each of 16S rRNA and 23S rRNA. The total draft genome consists of 6,159,766 bp with a G+C content of 40.9% and possesses an average coding sequence (CDS) length of 847 bp, an average intergenic length of 144 bp, and a protein coding density of 85.2%.

Further detailed analysis, including functional annotation, comparative genomics, and metabolic pathways analysis at the genome scale, is in process and will be included in our future publication.

### Nucleotide sequence accession numbers.

The draft genome sequence of *Clostridium ultunense* strain Esp has been deposited in the GenBank database with the accession numbers CARA01000001 to CARA01000281.
